# Improving Survival of Critical Care Patients With Coronavirus Disease 2019 in England: A National Cohort Study, March to June 2020*

**DOI:** 10.1097/CCM.0000000000004747

**Published:** 2020-10-26

**Authors:** John M. Dennis, Andrew P. McGovern, Sebastian J. Vollmer, Bilal A. Mateen

**Affiliations:** 1 Institute of Biomedical & Clinical Science, University of Exeter Medical School, Exeter, United Kingdom.; 2 The Alan Turing Institute, London, United Kingdom.; 3 Department of Statistics, University of Warwick, Coventry, United Kingdom.; 4 King’s College Hospital NHS Foundation Trust, Denmark Hill, London, United Kingdom.

**Keywords:** coronavirus infection, critical care, hospital mortality, public health surveillance, quality of healthcare

## Abstract

To measure temporal trends in survival over time in people with severe coronavirus disease 2019 requiring critical care (high dependency unit or ICU) management, and to assess whether temporal variation in mortality was explained by changes in patient demographics and comorbidity burden over time.

**Design:**

Retrospective observational cohort; based on data reported to the COVID-19 Hospitalisation in England Surveillance System. The primary outcome was in-hospital 30-day all-cause mortality. Unadjusted survival was estimated by calendar week of admission, and Cox proportional hazards models were used to estimate adjusted survival, controlling for age, sex, ethnicity, major comorbidities, and geographical region.

**Setting:**

One hundred eight English critical care units.

**Patients:**

All adult (18 yr +) coronavirus disease 2019 specific critical care admissions between March 1, 2020, and June 27, 2020.

**Interventions:**

Not applicable.

**Measurements and Main Results:**

Twenty-one thousand eighty-two critical care patients (high dependency unit *n* = 15,367; ICU *n* = 5,715) were included. Unadjusted survival at 30 days was lowest for people admitted in late March in both high dependency unit (71.6% survival) and ICU (58.0% survival). By the end of June, survival had improved to 92.7% in high dependency unit and 80.4% in ICU. Improvements in survival remained after adjustment for patient characteristics (age, sex, ethnicity, and major comorbidities) and geographical region.

**Conclusions:**

There has been a substantial improvement in survival amongst people admitted to critical care with coronavirus disease 2019 in England, with markedly higher survival rates in people admitted in May and June compared with those admitted in March and April. Our analysis suggests this improvement is not due to temporal changes in the age, sex, ethnicity, or major comorbidity burden of admitted patients.

## Abstract 2

Supplemental Digital Content is available in the text.

Recent national data from England suggests there has been an improvement in the survival of patients hospitalized with coronavirus disease 2019 (COVID-19) over recent months, with an estimated decline in the case-fatality rate of hospitalized patients from 6% in early April to 1.5% by mid-June (1). One potential explanation for this observation is a shift in the demographics of people admitted with COVID-19 toward younger and overall less comorbid individuals, resulting in improved survival. Another possibility is that expanded in-hospital testing to facilitate cohorting of potentially asymptomatic cases has led to increased identification of milder cases as the pandemic has progressed (2); in the early stages of the pandemic, it was not nationally mandated that all hospitalized patients were routinely tested. If the latter is a significant factor in the declining mortality, then we would expect mortality in the critical care setting to have remained relatively unchanged.

We sought to test whether the same trend of improving survival over time has been seen in people with severe COVID-19 requiring critical care (high dependency unit [HDU] or ICU) management, whether improving survival reflected changes in patient demographics, and whether time trends varied by demographics and geography in England.

## MATERIALS AND METHODS

### Data Source

We accessed national data from the COVID-19 Hospitalisation in England Surveillance System (CHESS) (3), a statutory collection of all individuals with confirmed or clinically presumed COVID-19 managed in HDU or ICU. CHESS data were extracted on July 27, 2020, at which time point 108 National Health Service (NHS) trusts had reported to CHESS (each NHS trust may comprise more than one hospital). Our study period was March 1 until June 27, meaning every patient had 30 days of potential follow-up available after being admitted to hospital. People 18–99 years old were eligible, pregnant women (*n* = 88) were excluded. Two cohorts were defined as follows: 1) all people admitted to HDU but never ICU and 2) all people admitted to ICU.

### Recorded Clinical Features

CHESS contains individual-level demographic characteristics: age, sex, ethnicity, admitting hospital, and recorded comorbidities (obesity; diabetes; asthma; chronic respiratory disease; chronic heart disease; hypertension; immunosuppression due to disease or treatment; chronic neurologic disease; chronic renal disease; and chronic liver disease). We coded ethnicity as White, Asian, Black, mixed, and other; categorized hospital centers by region: London, East of England, Midlands, North East, and Yorkshire, North West, South East, and South West; and recorded comorbidities as binary “No/Yes” variables.

### Statistical Analysis

The primary outcome was in-hospital all-cause mortality in the 30 days after hospital admission. Patients discharged alive or transferred prior to 30 days were assumed to be alive at 30 days (4). We estimated unadjusted survival for each week of the study period (17 wk in total, from the week of March 1, 2020, to the week of June 21, 2020) as 1–(number of deaths for patients admitted that week/total number of critical care admissions that week). We estimated adjusted hazard ratios (HRs) for mortality by week of admission (categorical variable, with the week starting March 29, the week of peak critical care admissions, used at the reference week) using separate Cox proportional hazards models for the HDU and ICU cohorts, adjusting for age (three-knot nonlinear restricted cubic spline), sex, ethnicity, comorbidities, and geographical region, with proportional hazards assumptions tested. For the ICU cohort, follow-up began at the date of ICU admission, with additional adjustment for the number of days from hospital to ICU admission (to capture possible changes in admission policy over time, for example, if there were delays in admitting patients to ICU earlier in the pandemic in the United Kingdom when concern over hospital capacity was greatest). After initial analysis modeling week of admission as a categorical variable, we ran additional models to estimate the average change in survival, per week, for the period of the week of March 29, 2020, to the week of June 21, 2020, by updating the same adjusted models but with week of admission fitted as a continuous linear term. As sensitivity analysis, we repeated models with NHS hospital trust included as a random effect. Analyses were conducted with R (Version 3.6.2 [5]), including use of the packages survival, rms and coxme.

### Ethics and Governance

The study was reviewed and approved by the Warwick Biosciences Research Ethics Committee (BSREC 119/19-20-V1.1) and sponsorship is being provided by University of Warwick (SOC.28/19-20).

## RESULTS

Twenty-one–thousand eighty-two individuals (HDU *n* = 15,367; ICU *n* = 5,715) were eligible for the study, of whom 5,484 (26.0%) died (HDU *n* = 3,438 [22.3%]; ICU *n* = 2,046 [35.8%]). Fifty-six percent of people admitted to HDU were male (mean age 70), compared with 71% male in ICU (mean age 58); full recorded characteristics are reported in **sTable 1** (Supplemental Digital Content 1, http://links.lww.com/CCM/F976).

**Figure 1.**
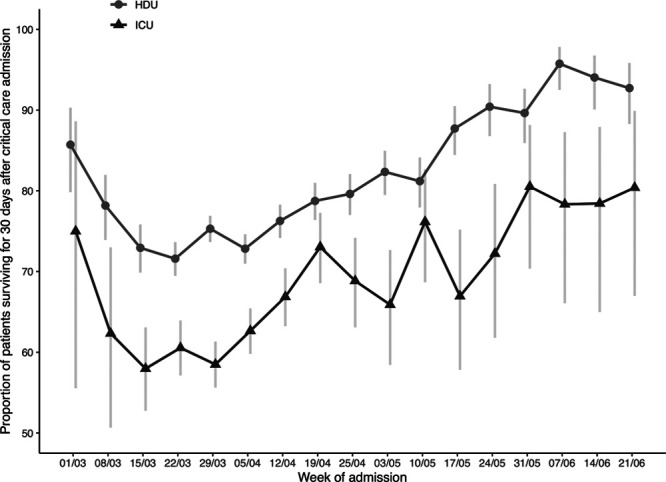
Unadjusted estimates of the proportion of patients surviving for 30 d after critical care admission, by week of admission, from the week of March 1, 2020, to the week of June 21, 2020. Point estimates are calculated as, for each week, 1–(number of deaths/number of hospital admissions). *Error bars* represent 95% CIs. HDU = high intensive unit.

Unadjusted mortality risk was highest for people admitted in early March in both HDU (28.4% for people admitted the week of March 22) and ICU (42.0% for those admitted during the week of March 15) (**Fig. 1**; for underlying data, **sTable 2**, Supplemental Digital Content 1, http://links.lww.com/CCM/F976). After the week of March 29, unadjusted risk of mortality improved for people admitted to both HDU and ICU.

**Figure 2.**
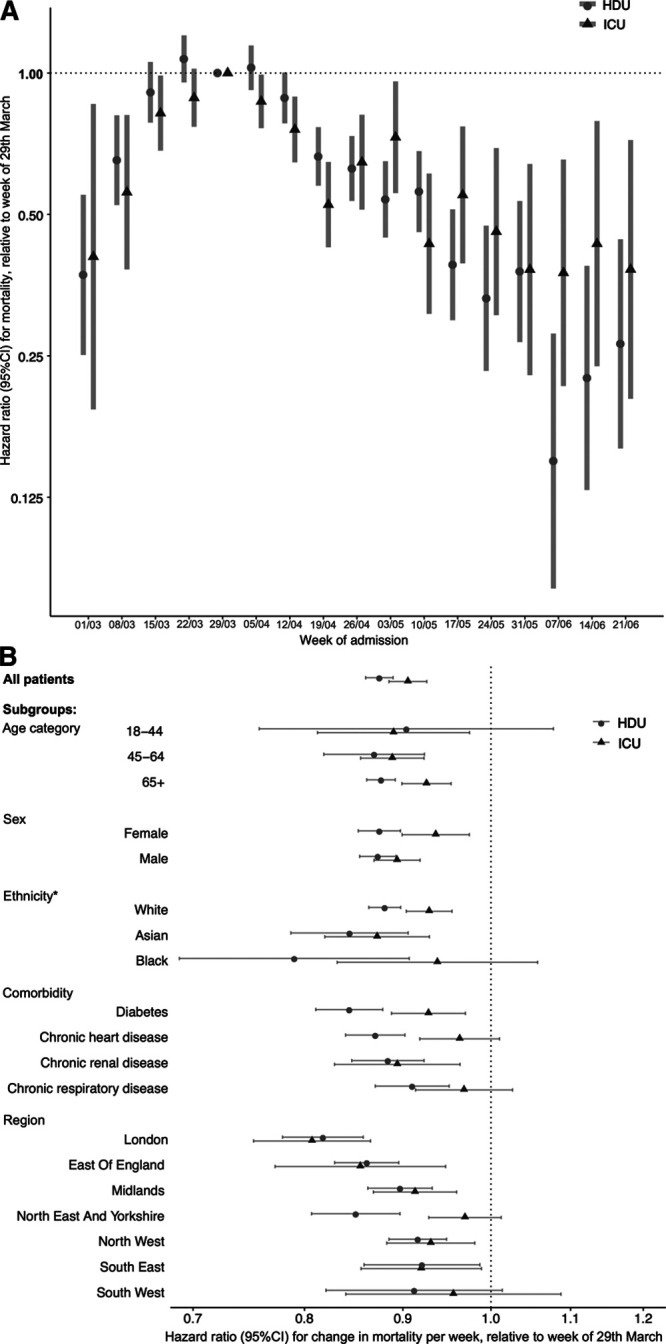
**A**, Adjusted hazard ratios (HRs) representing the relative change over time in-hospital mortality for people admitted to HDU and ICU with coronavirus disease 2019 (COVID-19) in England, up to the week of June 21, 2020. Bars represent 95% CIs. **B**, Relative mortality by week of hospital admission. An HR less than 1 reflects a lower mortality compared to patients admitted the week commencing March 29, 2020, which was used as the reference week as it was the peak week of COVID-19 admissions in COVID-19 Hospitalisation in England Surveillance System (CHESS). **B**, Average change in relative mortality per week, from the week of March 29, 2020, to the week of June 21, 2020, overall and for subgroups of patients. An HR less than 1 reflects a survival improvement. This figure shows that, for all patients, compared to people admitted during the week of March 29, 2020, for each subsequent week there was a 12.7% relative improvement in survival for high dependency unit (HDU) treated patients (adjusted HR for mortality 0.87 [95% CI 0.86-0.89]), and an 8.9% improvement in ICU treated patients (adjusted HR 0.91 [95% CI 0.89-0.93]). Similar improvements were seen across all age subgroups, in both men and women, and in people of White and Asian ethnicity. *HRs for mixed and other ethnicity defined subgroups not reported due to low patient numbers.

Improvements in survival after the week of March 29 were still observed after adjustment for patient characteristics (age, sex, ethnicity, and comorbidities) and geographical region (**Fig. 2*A***; for underlying data, **sTable 3**, Supplemental Digital Content 1, http://links.lww.com/CCM/F976). Relative to patients admitted during the week of March 29, survival improved by 12.7% per week in HDU (adjusted HR for mortality, 0.87; 95% CI, 0.86–0.89), and 8.9% in ICU (adjusted HR, 0.91; 95% CI, 0.89–0.93) up to the week of June 21, 2020 (Fig. 2*A*). Results were consistent with NHS hospital trust as an additional random effect.

The improvement in survival relative to the week of March 29 was consistent across subgroups defined by age, sex, ethnicity, and in people with major comorbidities (**Fig. 2*B***). Improvements were consistent by geographical region, except for in the North East and Yorkshire, where there was evidence of an improvement in HDU but not ICU (Fig. 2*B*).

## DISCUSSION

Our analysis, using a large national COVID-19 specific critical care database capturing information on hospital admissions up to July 27, 2020, shows a substantial recent improvement in survival for people admitted to critical care with COVID-19 in England, with markedly improved survival for people admitted after mid-April compared with earlier in the pandemic. Adjustment for age, sex, ethnicity, and major comorbidities suggests this improvement may not reflect a change in patient background characteristics. Similarly, adjustment for geographical region means temporal variation in critical care admissions across the country does not offer an explanation for our findings.

A survival improvement was observed for all regions in England in both HDU and ICU settings, although in the North East and Yorkshire, an improvement was only seen in patients admitted to HDU. A recent analysis of the CHESS dataset matched with national level audit data demonstrated a marked variation in survival by hospital trust for patients admitted to ICU that was not explained by severity profile (as assessed by Acute Physiology and Chronic Health Evaluation II score) of those admitted (6). The combination of these two results suggests that further study is warranted to understand differences in survival by hospital and geographical region across England.

In this study, we included patients from 108 out of 140 potential trusts in England reporting to CHESS by June 27, 2020. As such, there are unsurprisingly differences in the study population compared with that captured by the U.K.’s Intensive Care National Audit and Research Centre (ICNARC), the other national clinical audit of COVID-19 critical care admissions. ICNARC includes intensive care and combined intensive care/high dependency unit admissions (across Wales and Northern Ireland as well as England), while CHESS, a collection of all high dependency unit and ICU admissions, captures a broader inpatient population but is restricted to England. A comparison of the temporal trends observed in the CHESS ICU subgroup is similar to that of a recent (as of yet not peer-reviewed) report from ICNARC showing a substantial drop in odds of mortality over the course of the first wave (i.e., odds ratio 0.70 [0.61–0.81], for post-peak vs pre-pandemic, and 0.97 [0.86–1.08] for peak vs pre-pandemic) (7); however, our analysis extends this observation to the HDU setting thereby demonstrating that this effect is likely to be robust to the selection bias implicit in identifying candidates for intensivist care.

A potential limitation of this analysis is the possibility of reporting delays leading to incomplete ascertainment of death in more recent weeks, although this was mitigated by our study design, which focused on in-hospital mortality and ensured all eligible patients had at least 30 days follow-up available. While reporting delays might plausibly lead to under-ascertainment of mortality for people admitted in May, a clear mortality improvement was also observed in April. As CHESS is a daily mandatory collection for all hospitals in England, we would expect reasonably accurate and timely capture of in-hospital mortality, and so feel it is unlikely our findings simply reflect reporting delays.

Temporal changes in COVID-19 disease severity at admission, patient selection for critical care management, critical care treatment, hospital capacity, and COVID-19 testing all offer potential explanations for our findings. Of particular note regarding treatment, the RECOVERY trial began nationwide recruitment in early April, and included dexamethasone which was later shown to have mortality-specific, or length of intensive treatment unit admission-specific benefits, as well as azithromycin, tocilizumab, and convalescent plasma (8). Randomised Evaluation of COVID-19 Therapy (RECOVERY) did not include remdesivir, also shown to have therapeutic benefit in COVID-19 patients, but did not exclude patients receiving remdesivir or other potential COVID-19 treatments from the trial itself (9). It is, therefore, plausible recruitment to RECOVERY might partially explain improved patient outcomes, although we lacked data on medications administered in hospital to evaluate this. Regarding capacity, it has been shown that bed saturation across England was at its highest in early April and then progressively improved over the course of the first wave of the pandemic (10). Therefore, the observed time trend could be a manifestation of the well-established decline in patient-specific outcomes often observed at “unsafe” occupancy levels (11). Further interrogation of possible quality-of-care based explanations is required.

In conclusion, there has been a substantial mortality improvement in people admitted to critical care with COVID-19 in England, with markedly lower mortality in people admitted in mid-April and May compared with earlier in the pandemic. This trend remains after adjustment for patient demographics and comorbidities, suggesting this improvement is not due to changing patient characteristics. Possible causes include the introduction of effective treatments as part of the RECOVERY trial, improved physician understanding of the disease process, and a falling critical care burden.

## Supplementary Material


