# Supplementary material for: Improving Survival of Critical Care Patients With Coronavirus Disease 2019 in England: A National Cohort Study, March to June 2020*
Source: Crit Care Med. 2020 Oct 26;49(2):209–14. doi: 10.1097/CCM.0000000000004747 (PMC7803441; doi:10.1097/CCM.0000000000004747)
Supplement: Supplementary file 1 [file ccm-49-0209-s001.docx]

**Supplementary Material for Improving survival of critical care patients with COVID-19 in England: A national cohort study, March to June 2020**

***Authors:*** *John M. Dennis, Andrew P. McGovern, Sebastian J. Vollmer, & Bilal A. Mateen*

**sTable 1: Recorded characteristics of HDU and ICU cohorts.** Data are N (%) unless stated.

|  | **HDU (n=15,367)** | **ICU (n=5,715)** |
| --- | --- | --- |
| **Age (mean [SD])** | 70 (17) | 58 (13) |
| **Sex** | | |
| Female | 6,755 (44.0) | 1,674 (29.3) |
| Male | 8,612 (56.0) | 4,041 (70.7) |
| **Ethnicity*** | | |
| White | 8,948 (77.7) | 3,142 (64.3) |
| Asian | 1,240 (10.8) | 848 (17.4) |
| Black | 724 (6.3) | 378 (7.7) |
| Mixed | 76 (0.7) | 143 (2.9) |
| Other | 523 (4.5) | 375 (7.7) |
| **Obesity**** | | |
| Non-obese | 5,450 (75.6) | 2,068 (47.7) |
| Obese | 1,763 (24.4) | 2,270 (52.3) |
| **Comorbidity** | | |
| Diabetes | 2,313 (15.1) | 1,451 (25.4) |
| Chronic renal disease | 1,472 (9.6) | 410 (7.2) |
| Chronic respiratory disease | 1,225 (8.0) | 514 (9.0) |
| Chronic heart disease | 2,099 (13.7) | 621 (10.9) |
| Asthma | 950 (6.2) | 709 (12.4) |
| Hypertension | 3,698 (24.1) | 2,069 (36.2) |
| Chronic liver disease | 250 (1.6) | 133 (2.3) |
| Chronic neurological disease | 1,196 (7.8) | 232 (4.1) |
| Immunosuppressive disease | 285 (1.9) | 185 (3.2) |
| **Region***** |  |  |
| East of England | 2125 (13.8) | 419 (7.3) |
| London | 4267 (27.8) | 1413 (24.7) |
| Midlands | 2885 (18.8) | 770 (13.5) |
| North East & Yorkshire | 1740 (11.3) | 857 (15.0) |
| North West | 1942 (12.6) | 701 (12.3) |
| South East | 1071 (7.0) | 822 (14.4) |
| South West | 385 (2.5) | 364 (6.4) |

*Ethnicity not recorded for 3,856 HDU patients and 829 ICU patients. **Obesity not recorded for 8,144 HDU patients and 1,377 ICU patients. ***Region not available for 952 HDU patients and 369 ICU patients.

**sTable 2: Unadjusted estimates of the proportion of patients surviving for 30-days after critical care admission, by week of admission, from the week of 1st March, 2020 to the week of 21st June, 2020.** Survival estimates are calculated as, for each week, 1-(number of deaths/number of hospital admissions) as every patient had 30 days follow-up

**A) HDU**

| **Week of admission** | **Number of patients admitted** | **Number of patients dying within 30 days of admission** | **Proportion surviving (95% CI)** |
| --- | --- | --- | --- |
| 01-Mar | 182 | 26 | 85.7 (79.8, 90.3) |
| 08-Mar | 417 | 91 | 78.2 (73.9, 82.0) |
| 15-Mar | 872 | 236 | 72.9 (69.9, 75.8) |
| 22-Mar | 1809 | 514 | 71.6 (69.5, 73.6) |
| 29-Mar | 2709 | 669 | 75.3 (73.7, 76.9) |
| 05-Apr | 2311 | 628 | 72.8 (71.0, 74.6) |
| 12-Apr | 1673 | 397 | 76.3 (74.2, 78.3) |
| 19-Apr | 1242 | 264 | 78.7 (76.4, 81.0) |
| 26-Apr | 986 | 201 | 79.6 (77.0, 82.1) |
| 03-May | 759 | 134 | 82.3 (79.5, 85.0) |
| 10-May | 622 | 117 | 81.2 (77.9, 84.1) |
| 17-May | 464 | 57 | 87.7 (84.4, 90.5) |
| 24-May | 334 | 32 | 90.4 (86.8, 93.2) |
| 31-May | 328 | 34 | 89.6 (85.9, 92.6) |
| 07-Jun | 235 | 10 | 95.7 (92.5, 97.8) |
| 14-Jun | 218 | 13 | 94.0 (90.1, 96.8) |
| 21-Jun | 206 | 15 | 92.7 (88.3, 95.8) |

**B) ICU**

| **Week of admission** | **Number of patients admitted** | **Number of patients dying within 30 days of admission** | **Proportion surviving (95% CI)** |
| --- | --- | --- | --- |
| 01-Mar | 28 | 7 | 75.0 (55.5, 88.6) |
| 08-Mar | 77 | 29 | 62.3 (50.7, 73.0) |
| 15-Mar | 364 | 153 | 58.0 (52.8, 63.1) |
| 22-Mar | 801 | 316 | 60.5 (57.1, 63.9) |
| 29-Mar | 1166 | 484 | 58.5 (55.6, 61.3) |
| 05-Apr | 1154 | 431 | 62.7 (59.8, 65.4) |
| 12-Apr | 670 | 222 | 66.9 (63.2, 70.4) |
| 19-Apr | 408 | 110 | 73.0 (68.5, 77.3) |
| 26-Apr | 276 | 86 | 68.8 (63.1, 74.2) |
| 03-May | 173 | 59 | 65.9 (58.4, 72.7) |
| 10-May | 151 | 36 | 76.2 (68.7, 82.7) |
| 17-May | 118 | 39 | 66.9 (57.8, 75.2) |
| 24-May | 90 | 25 | 72.2 (61.8, 80.9) |
| 31-May | 77 | 15 | 80.5 (70.4, 88.2) |
| 07-Jun | 60 | 13 | 78.3 (66.1, 87.3) |
| 14-Jun | 51 | 11 | 78.4 (65.0, 87.9) |
| 21-Jun | 51 | 10 | 80.4 (67.0, 89.9) |

**sTable 3: Hazard ratios (95% confidence intervals) for full covariate set in multivariable analysis of the primary outcome of 30 day all-cause in-hospital mortality for each study cohort.**

|  |  | **HDU cohort**  **(n=15,367)** | **ICU cohort**  **(n=5,715)** |
| --- | --- | --- | --- |
| **Week of admission (categorical variable)** | | | |
| 01-Mar |  | 0.37 (0.25, 0.55) | 0.41 (0.19, 0.86) |
| 08-Mar |  | 0.65 (0.52, 0.81) | 0.56 (0.38, 0.81) |
| 15-Mar |  | 0.91 (0.78, 1.06) | 0.82 (0.68, 0.99) |
| 22-Mar |  | 1.07 (0.95, 1.20) | 0.89 (0.77, 1.02) |
| 29-Mar |  | 1.00 (ref) | 1.00 (ref) |
| 05-Apr |  | 1.03 (0.92, 1.14) | 0.87 (0.76, 0.99) |
| 12-Apr |  | 0.89 (0.78, 1.00) | 0.76 (0.65, 0.89) |
| 19-Apr |  | 0.66 (0.58, 0.77) | 0.52 (0.43, 0.65) |
| 26-Apr |  | 0.63 (0.53, 0.73) | 0.65 (0.51, 0.81) |
| 03-May |  | 0.54 (0.45, 0.65) | 0.73 (0.56, 0.96) |
| 10-May |  | 0.56 (0.46, 0.68) | 0.43 (0.31, 0.61) |
| 17-May |  | 0.39 (0.30, 0.51) | 0.55 (0.39, 0.77) |
| 24-May |  | 0.33 (0.23, 0.47) | 0.46 (0.31, 0.69) |
| 31-May |  | 0.38 (0.27, 0.53) | 0.38 (0.23, 0.64) |
| 07-Jun |  | 0.15 (0.08, 0.28) | 0.38 (0.22, 0.65) |
| 14-Jun |  | 0.22 (0.13, 0.39) | 0.43 (0.24, 0.79) |
| 21-Jun |  | 0.27 (0.16, 0.44) | 0.38 (0.20, 0.72) |
| **Age** | | | |
| Age spline |  | 0.96 (0.96, 0.97) | 1.04 (1.03, 1.05) |
| Age spline' |  | 1.27 (1.18, 1.36) | 1.01 (1.00, 1.02) |
| **Sex** | | | |
| Female |  | 1·00 (ref) | 1·00 (ref) |
| Male |  | 1.24 (1.07, 1.44) | 1.06 (0.96, 1.17) |
| **Ethnicity** | | | |
| White |  | 1.00 (ref) | 1.00 (ref) |
| Asian |  | 1.24 (1.07, 1.44) | 1.19 (1.04, 1.35) |
| Black |  | 1.13 (0.93, 1.37) | 1.12 (0.94, 1.35) |
| Mixed |  | 1.33 (0.80, 2.22) | 1.10 (0.81, 1.48) |
| Other |  | 0.80 (0.61, 1.05) | 0.91 (0.75, 1.12) |
| Missing |  | 0.88 (0.80, 0.96) | 0.88 (0.77, 1.01) |
| **Obesity** | | | |
| Non-obese (BMI<30) |  | 1.00 (ref) | 1.00 (ref) |
| Obese (BMI≥30) |  | 0.85 (0.75, 0.96) | 1.44 (1.28, 1.61) |
| Missing |  | 0.81 (0.74, 0.88) | 0.94 (0.85, 1.05) |
| **Comorbidity (Yes vs. No)** | | | |
| Type 2 diabetes |  | 1.21 (1.10, 1.32) | 1.21 (1.09, 1.35) |
| Chronic renal disease |  | 1.21 (1.09, 1.34) | 1.33 (1.14, 1.54) |
| Chronic respiratory disease |  | 1.35 (1.21, 1.50) | 1.16 (1.00, 1.34) |
| Chronic heart disease |  | 1.17 (1.06, 1.28) | 1.20 (1.05, 1.37) |
| Asthma |  | 0.97 (0.84, 1.12) | 0.92 (0.79, 1.06) |
| Hypertension |  | 0.95 (0.88, 1.04) | 1.07 (0.97, 1.19) |
| Chronic liver disease |  | 1.12 (0.86, 1.47) | 1.00 (0.74, 1.36) |
| Chronic neurological disease |  | 1.29 (1.16, 1.44) | 1.28 (1.04, 1.58) |
| Immunosuppressive disease |  | 1.23 (0.99, 1.52) | 1.22 (0.96, 1.53) |
| **Time in hospital** | | | |
| Days from hospital to ICU admission (per day increase) |  | NA | 1.01 (1.00, 1.01) |
| **Region** |  |  |  |
| East of England |  | 1.66 (1.48, 1.87) | 0.85 (0.70, 1.02) |
| London |  | 1.00 (ref) | 1.00 (ref) |
| Midlands |  | 0.91 (0.80, 1.03) | 0.94 (0.81, 1.09) |
| North East & Yorkshire |  | 1.20 (1.06, 1.37) | 0.96 (0.83, 1.11) |
| North West |  | 1.15 (1.01, 1.30) | 0.80 (0.68, 0.95) |
| South East |  | 0.86 (0.73, 1.00) | 0.66 (0.56, 0.76) |
| South West |  | 1.49 (1.21, 1.84) | 0.61 (0.49, 0.76) |
| Missing |  | 0.34 (0.26, 0.45) | 0.61 (0.49, 0.77) |
